# Presynaptic GABA_B_ Receptors Regulate Hippocampal Synapses during Associative Learning in Behaving Mice

**DOI:** 10.1371/journal.pone.0148800

**Published:** 2016-02-05

**Authors:** M. Teresa Jurado-Parras, José M. Delgado-García, Raudel Sánchez-Campusano, Martin Gassmann, Bernhard Bettler, Agnès Gruart

**Affiliations:** 1 Division of Neurosciences, Pablo de Olavide University, 41013, Seville, Spain; 2 Department of Biomedicine, University of Basel, 4056, Basel, Switzerland; IGBMC/ICS, FRANCE

## Abstract

GABA_B_ receptors are the G-protein-coupled receptors for GABA, the main inhibitory neurotransmitter in the central nervous system. Pharmacological activation of GABA_B_ receptors regulates neurotransmission and neuronal excitability at pre- and postsynaptic sites. Electrophysiological activation of GABA_B_ receptors in brain slices generally requires strong stimulus intensities. This raises the question as to whether behavioral stimuli are strong enough to activate GABA_B_ receptors. Here we show that GABA_B1a_^-/-^ mice, which constitutively lack presynaptic GABA_B_ receptors at glutamatergic synapses, are impaired in their ability to acquire an operant learning task. *In vivo* recordings during the operant conditioning reveal a deficit in learning-dependent increases in synaptic strength at CA3-CA1 synapses. Moreover, GABA_B1a_^-/-^ mice fail to synchronize neuronal activity in the CA1 area during the acquisition process. Our results support that activation of presynaptic hippocampal GABA_B_ receptors is important for acquisition of a learning task and for learning-associated synaptic changes and network dynamics.

## Introduction

GABA_B_ receptors regulate neuronal excitability and synaptic transmission in the brain. Axonal GABA_B_ receptors inhibit voltage-activated Ca^2+^-channels to reduce the release of GABA, glutamate, and other neurotransmitters [1]-[2]. Dendritic GABA_B_ receptors activate Kir3 channels and inhibit neuronal activity by local shunting and by generating hyperpolarizing postsynaptic potentials. Consistent with the remote location of GABA_B_ receptors in relation to the sites of GABA release [3], electrophysiological activation of GABA_B_ receptors in brain slices typically requires strong stimulus intensities and pooling of synaptically released GABA from many interneurons [4]-[7]. This suggests that GABA_B_ receptors are primarily activated during rhythmic cortical or hippocampal network activity, when ensembles of GABAergic neurons fire in synchrony [5]-[7]. It is still poorly understood whether GABA_B_ receptors in the brain are mostly involved in homeostatic processes or whether they are activated in response to sensory inputs and behavioral tasks [8].

GABA_B_ receptors are composed of principal and auxiliary subunits [2]. The principal subunits GABA_B1a_, GABA_B1b_, and GABA_B2_ form fully functional heteromeric GABA_B(1a,2)_ and GABA_B(1b,2)_ receptors with indistinguishable properties in heterologous cells. The subunit isoform GABA_B1a_ contains a targeting motif in its primary sequence that traffics receptors to axon terminals [9]. In contrast, the subunit isoform GABA_B1b_ traffics receptors to dendritic sites. The auxiliary subunits KCTD8, -12, -12b and -16 are cytoplasmic proteins that constitutively bind to GABA_B2_ and modulate kinetic properties of the receptor response [10]-[11]. The *in vivo* roles of GABA_B(1a,2)_ and GABA_B(1b,2)_ receptors were addressed using GABA_B1a_^-/-^ and GABA_B1b_^-/-^ mice that lack one or the other subunit isoform [12]. These mice exhibit distinct behavioral phenotypes, consistent with GABA_B(1a,2)_ and GABA_B(1b,2)_ receptors conveying non-redundant pre- and postsynaptic functions [2], [13]. GABA_B1a_^-/-^ mice exhibited hippocampus-dependent cognitive deficits [12], impaired emotional learning [14], fragmented sleep [15], and infrequent seizures at an advanced age [15]. These phenotypes revealed that the genetic absence of presynaptic GABA_B(1a,2)_ receptors interferes with mnemonic processes and eventually precipitates seizures. However, whether GABA_B(1a,2)_ receptors are involved in the regulation of synaptic strength and network dynamics during the acquisition and performance of a learning task is still unclear.

Here, we used GABA_B1a_^-/-^ mice to address whether the lack of presynaptic GABA_B_ receptors influences changes in synaptic strength and network synchronizations during a learning task. Specifically, we recorded changes in synaptic strength [16]-[17] and oscillatory dynamics [17] at hippocampal CA3-CA1 synapses by continuously recording evoked field excitatory postsynaptic potentials (fEPSPs) during the acquisition and performance of an operant conditioning task. Operant conditioning is a type of associative learning involving the activity of many cortical circuits, including the hippocampus [17]-[18]. We found that learning-dependent changes in synaptic strength and network synchronizations in behaving GABA_B1a_^-/-^ mice are impaired, while basic synaptic properties are normal. Our results support that learning processes and learning-associated network synchronizations depend on the presence of presynaptic GABA_B_ receptors in the hippocampus.

## Methods

### Experimental Animals

All experiments were carried out with male GABA_B1a_^-/-^ and wild-type (WT) littermate BALB/c mice [12]. Mice were 4 ± 1 months old at the beginning of the experiments and were kept on a 12-h light/dark cycle at a constant temperature (21 ± 1°C) and humidity (50 ± 7%) until surgery. Mice had *ad libitum* access to chow and water.

### Ethics Statement

The experiments were performed during the light cycle according to European Union Council (2010/63/EU) and Spanish (BOE 34/11370-421, 2013) guidelines. All experiments were approved by the local Ethics Committee (Permit Number 01/11) of the Pablo de Olavide University (Seville, Spain).

### Surgery

Mice were anesthetized with 0.8–3% halothane delivered from a calibrated Fluotec 5 (Fluotec-Ohmeda, Tewksbury, MA) vaporizer at a flow rate of 1–2 L/min oxygen. Mice were implanted with bipolar stimulating electrodes aimed at the right Schaffer collateral-commissural pathway of the dorsal hippocampus (2 mm lateral and 1.5 mm posterior to bregma, depth from brain surface 1.0–1.5 mm) [19] and with two recording electrodes aimed at the ipsilateral *stratum radiatum* underneath the CA1 area (1.2 mm lateral and 2.2 mm posterior to bregma; depth from brain surface 1.0–1.5 mm) [19]. The location of the electrodes was verified histologically (see below) and from the profiles of collected fEPSPs. This showed that most electrodes were located in the *stratum radiatum* and few electrodes near the pyramidal cell layer. From each animal we selected the recording electrode providing better defined fEPSP responses. These electrodes were made from Teflon-coated tungsten wire (50 μm, Advent Research Materials Ltd., Eynsham, England). The final position of hippocampal electrodes was determined using as a guide the field potential depth profile evoked by paired (40 ms of interval) pulses presented to the Schaffer collateral pathway. A bare silver wire (0.1 mm) was affixed to the skull as a ground. The four wires were connected to a 4-pin socket and the latter was fixed to the skull with the help of two small screws and dental cement [16]. Ten mice were used per experimental group.

### Recording and Stimulation Procedures

fEPSPs were recorded with Grass P511 differential amplifiers through a high-impedance probe (2 × 10^12^ Ω, 10 pF). Electrical stimuli presented to Schaffer collaterals consisted of 100 μs, square, biphasic pulses presented alone, paired, or in trains. Stimulus intensities ranged from 0.02 mA to 0.4 mA for the construction of the input/output curves. For paired-pulse facilitation, the stimulus intensity was set well below the threshold for evoking a population spike, usually 35% of the intensity necessary for evoking a maximum fEPSP response [20]. Paired pulses were presented at six (10, 20, 40, 100, 200, and 500 ms) different pulse intervals.

For LTP induction, the stimulus intensity was set at 35% of the intensity necessary to evoke a maximum fEPSP response. An additional criterion for selecting the stimulus intensity for LTP induction was that a second stimulus, presented 40 ms after a conditioning pulse, evoked a larger (> 20%) synaptic field potential than the first stimulus [21]. After 15 min of baseline recording at 0.05 Hz, each animal was presented with a HFS protocol consisting of five trains (200 Hz, 100 ms) of pulses at a rate of 1/s. This protocol was applied 6 times at intervals of 1 min. The evolution of fEPSPs after the HFS protocol was followed for 60 min at 0.05 Hz. Additional recording sessions (30 min) were carried out during the following four days. Further experimental details can be found elsewhere [16], [22].

### Operant Conditioning

GABA_B1a_^-/-^ mice at the age of operant conditioning show normal locomotor activity and have normal body weight, indicating the absence of overt motor deficits or motivational changes that could influence performance [2], [14]. Training and testing took place in basic Skinner box modules (n = 3) measuring 12.5 cm × 13.5 cm × 18.5 cm (MED Associates, St. Albans, VT, USA). The operant chambers were housed within a sound-attenuating chamber (90 cm × 55 cm × 60 cm), which was constantly illuminated (19 W lamp) and exposed to a 45 dB white noise (Cibertec, S.A., Madrid, Spain). Each Skinner box was equipped with a food dispenser from which pellets (MLabRodent Tablet, 20 mg; Test Diet, Richmond, IN, USA) could be delivered by pressing a lever. Before training, mice were handled daily for 7 days and food-deprived to 75–85% of their free-feeding weight [23]-[24]. Training took place for 20 min during successive days, in which mice were shaped to press the lever to receive pellets from the food tray using a fixed-ratio (1:1) schedule. The start and end of each session was indicated by a tone (2 kHz, 200 ms, 70 dB) provided by a loudspeaker located in the recording chamber. Animals were maintained on the 1:1 schedule until they reached the criterion and obtained ≥ 20 pellets in each of 2 successive sessions. WT mice typically reached this criterion after 4–6 days of training [24].

We recorded fEPSPs evoked at the CA3-CA1 synapse across the training (fixed ratio 1:1) sessions. Baseline recordings were collected at session 0 with the animal placed in the same Skinner box but in the absence of the lever and the feeder. Here again, the stimulus intensity was set at 35% of the intensity necessary for evoking a maximum fEPSP response. LFP analyses were carried out using recordings collected in the absence of Schaffer-collateral stimulation [17].

Mice that reached the criterion for the fixed-ratio 1:1 schedule in ≤ 6 sessions were further conditioned using a light/dark protocol for 6 additional days. In this protocol, only lever presses during the light period (20 s) were reinforced with a pellet. Lever presses performed during the dark period (20 ± 10 s) were not reinforced and restarted the dark protocol for an additional random (1–10 s) time. The light/dark coefficient was calculated as follows: (number of lever presses during the light period—number of lever presses during the dark period) / total number of lever presses. In this case, the criterion was to reach a 0 performance (i.e., an equal number of lever presses during the light and the dark periods) for 2 successive sessions.

Conditioning programs, lever presses, and delivered reinforcements were controlled and recorded by a computer, using a MED-PC program (MED Associates, St. Albans, VT, USA). All operant sessions including brain stimulation and/or LFP and fEPSP recordings were filmed with a synchronized video capture system (Sony HDR-SR12E, Tokyo, Japan).

### Drugs

To study the propensity of WT and GABA_B1a_^-/-^ mice to generate convulsive seizures, we injected them (i.p.) with the AMPA/kainate receptor agonist kainate (8 mg/kg; Sigma, Saint Louis, MO, USA) dissolved in 0.1 M phosphate buffered saline (PBS) pH = 7.4 [25]. The electrocorticographic activity of the hippocampal pyramidal CA1 area was recorded before and up to 1 h after the injection. Animals were presented with a stimulus session (five 100-millisecond trains of pulses at a rate of 200 Hz) 30 min after the injection.

### Histology

Once experiments were finished, mice were deeply re-anesthetized (sodium pentobarbital, 50 mg/kg) and perfused transcardially with saline and 4% phosphate-buffered paraformaldehyde. Their brains were removed, postfixed overnight at 4°C, and cryoprotected in 30% sucrose in PBS. Sections were obtained in a microtome (Leica, Wetzlar, Germany) at 50 μm. Selected sections including the dorsal hippocampus were mounted on gelatinized glass slides and stained using the Nissl technique with 0.1% toluidine blue to determine the location of stimulating and recording electrodes.

### Data Collection and Analysis

LFP, fEPSP, and 1-volt rectangular pulses corresponding to lever presses, pellet delivery, and brain stimulation, were stored digitally on a computer through an analog/digital converter (CED 1401 Plus, Cambridge, England). Data were analyzed off-line for quantification of each animal's performance in the Skinner box, LFP, and fEPSP with the Spike 2 (CED) program. The slope of evoked fEPSPs was computed as the first derivative (volts/s) of fEPSP recordings (volts). Five successive fEPSPs were averaged, and the mean value of the slope during the rise-time period (i.e., the period of the slope between the initial 10% and the final 10% of the fEPSP) was determined. Computed results were processed for statistical analysis using the IBM SPSS Statistics 18.0 software (IBM, Armonk, NY, USA).

For LFP analysis we used data collected across learning sessions until animals reached the criterion in the fixed-ratio (1:1) schedule or from selective recordings carried out to determine the kainate effects. As described by some of us [17], the analytical procedures, including the frequency domain (using the fast Fourier transform) and analyses of the LFP recordings, as well as the quantification and representation scripts of the power spectral density (PSD plots), were developed with the help of MATLAB routines (The MathWorks, Natick, MA, USA). The purpose of estimating the PSD was to detect any periodicity in the LFP data, by observing peaks at the frequencies corresponding to these periodicities. We selected the following frequency bands: delta, 1–3 Hz; low theta, 3–8 Hz; high theta 8–12 Hz; beta, 12–30 Hz; and HFS, 30–100 Hz. The algorithm included the analysis of mean values of the spectral powers between the different frequency bands inside each epoch, and the analysis of mean values of the spectral powers for the same frequency band between the different epochs. The same procedure was carried out for the peak values of the spectral powers inside each frequency band [17].

Data are always represented as the mean ± SEM. Statistical significance of differences between groups was inferred by one-way ANOVA and ANOVA for repeated measures (data by groups), with a contrast analysis (Dunnett's Post Hoc test) for a further study of significant differences. Statistical significance was set at *P* < 0.05.

## Results

### GABA_B1a_^-/-^ Mice Exhibit Deficits in Operant Conditioning and Learning-Dependent Changes in Synaptic Strength

**Fig 1:**
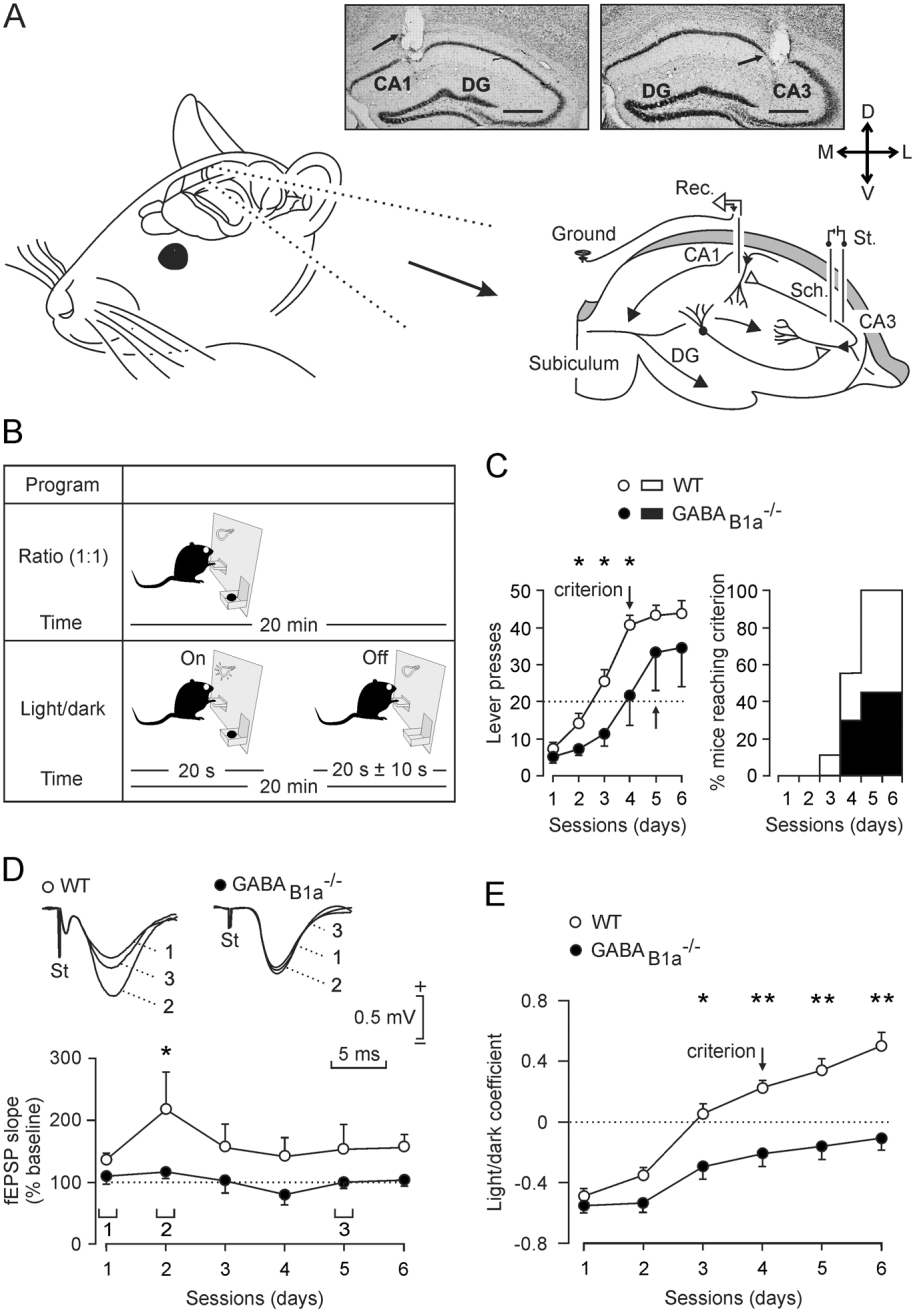
GABA_B1a_^-/-^ mice exhibit deficits in executing an operant conditioning task. *(A*) Mice were chronically implanted with stimulating (St.) and recording (Rec.) electrodes in the Schaffer collaterals/commissural pathway (Sch.) and the CA1 area, respectively. Representative photomicrographs illustrating the location (arrows) of recording (left) and stimulating (right) electrodes in post-experimental tissue are shown at the top. Calibration bars: 500 μm. (*B*) Mice were trained in a Skinner box to press a lever to obtain a food pellet. For operant conditioning we used two paradigms of increasing difficulty. In the first paradigm (Fixed-ratio of 1:1) mice had to press the lever ≥ 20 times per 20 min session for two successive sessions to successfully complete the task (criterion). In the second paradigm (Light/Dark), lever presses were rewarded only when a light bulb was switched on. Lever presses while the bulb was off were punished with a time penalty of 10 s during which the bulb would not turn on. In this case the animal had to press the lever at least the same number of times during the light and the dark periods for two successive sessions to successfully complete the task (criterion). (*C*) Performance of mice during 6 days of training with the 1:1 ratio schedule. WT mice (open circles) pressed the lever significantly more (*P* ≤ 0.02) and reached the criterion (arrow) in fewer sessions than GABA_B1a_^-/-^ mice (closed circles). All WT mice but only 40% of the GABA_B1a_^-/-^ mice successfully completed the task within 6 sessions (right panel). (*D*) Analysis of evoked fEPSP at the CA3-CA1 synapses during operant conditioning. The fEPSP slopes of WT mice were significantly (*P* < 0.02) larger than those of GABA_B1a_^-/-^ mice during the 2^nd^ session. Representative fEPSPs recorded during the indicated sessions (1 and 2) are shown at the top. ***E***, Performance of mice during 6 days of training in the light/dark test. WT mice performed significantly better than GABA_B1a_^-/-^ mice (*, *P* = 0.03; **, *P* ≤ 0.006) and reached the criterion by the 4^th^ session (arrow). GABA_B1a_^-/-^ mice failed to reach the criterion. The light/dark coefficient was calculated as follows: (number of lever presses during the light period—number of lever presses during the dark period) / total number of lever presses.

For *in vivo* electrophysiological analysis of behaving mice we chronically implanted stimulating and recording electrodes in the Schaffer collaterals/commissural pathway and in the hippocampal CA1 area, respectively (Fig 1A). The mice were then trained in a Skinner box to obtain a food pellet every time they press a lever in daily sessions of 20 min (fixed ratio of 1:1, Fig 1B). The mice successfully completed the task when pressing the lever ≥ 20 times in 2 successive sessions. It is established that the hippocampus participates in the acquisition and storage of this type of associative learning [17], [22], [24]. Littermate WT mice successfully completed the task in significantly fewer sessions than GABA_B1a_^-/-^ mice [F_(1,14)_ = 168.633; *P* < 0.001; Fig 1C, left]. All WT mice completed the task within 6 sessions while only 40% of the GABA_B1a_^-/-^ mice were successful (Fig 1C, right). In accordance with a previous study [17] and in order to study changes in synaptic strength at the CA3-CA1 synapse during the acquisition process, we evoked fEPSPs (≤ 3 times/min) at the moment the animal was approaching the lever (Fig 1D). The analysis of evoked fEPSPs at the CA3-CA1 synapse indicated that WT but not GABA_B1a_^-/-^ mice exhibited a significant increase in synaptic strength during the acquisition process [F_(1,14)_ = 1.131; *P* = 0.027; Fig 1D]. A separate analysis of GABA_B1a_^-/-^ mice that did or did not reach the selected criterion indicates that both subgroups did not exhibit a significant increase (*P* = 0.457) in fEPSP slopes during the acquisition sessions. Therefore, successful completion of the learning task by the GABA_B1a_^-/-^ mice does not predict an increase in synaptic strength.

Mice that successfully reached the above criterion within 6 days were subjected to a more complex operant conditioning task. Pressing the lever was rewarded with a food pellet only during periods of 20 s in which a light bulb above the lever was switched on (light/dark, Fig 1B). All WT littermate mice successfully reached the selected criterion [see Methods; F_(1,15)_ = 7.740; *P* = 0.014] by the 4th conditioning session, while GABA_B1a_^-/-^ mice were unsuccessful in completing the task (Fig 1E). No significant differences were observed in the slopes of fEPSPs in the two groups of mice during the training sessions (not illustrated). A possible explanation for the absence of plasticity changes during the light/dark test is that, as previously reported [17], changes in synaptic strength for appetitive behaviors (such as rewarded lever presses) during operant conditioning only take place during the early phase of the acquisition process.

Since GABA_B1a_^-/-^ mice did not exhibit any overt motor or motivational impairment [2] the above findings cannot be ascribed to any specific difficulty to move around in the Skinner box or to any evident hyperactivity or motor inactivity. In conclusion, these data show that the lack of presynaptic GABA_B_ receptors results in a significant deficit in the ability to learn an operant task and prevents learning-dependent changes in synaptic strength at CA3-CA1 synapses.

### GABA_B1a_^-/-^ Mice Fail to Synchronize Neuronal Activity in the CA1 Area during Operant Conditioning

**Fig 2:**
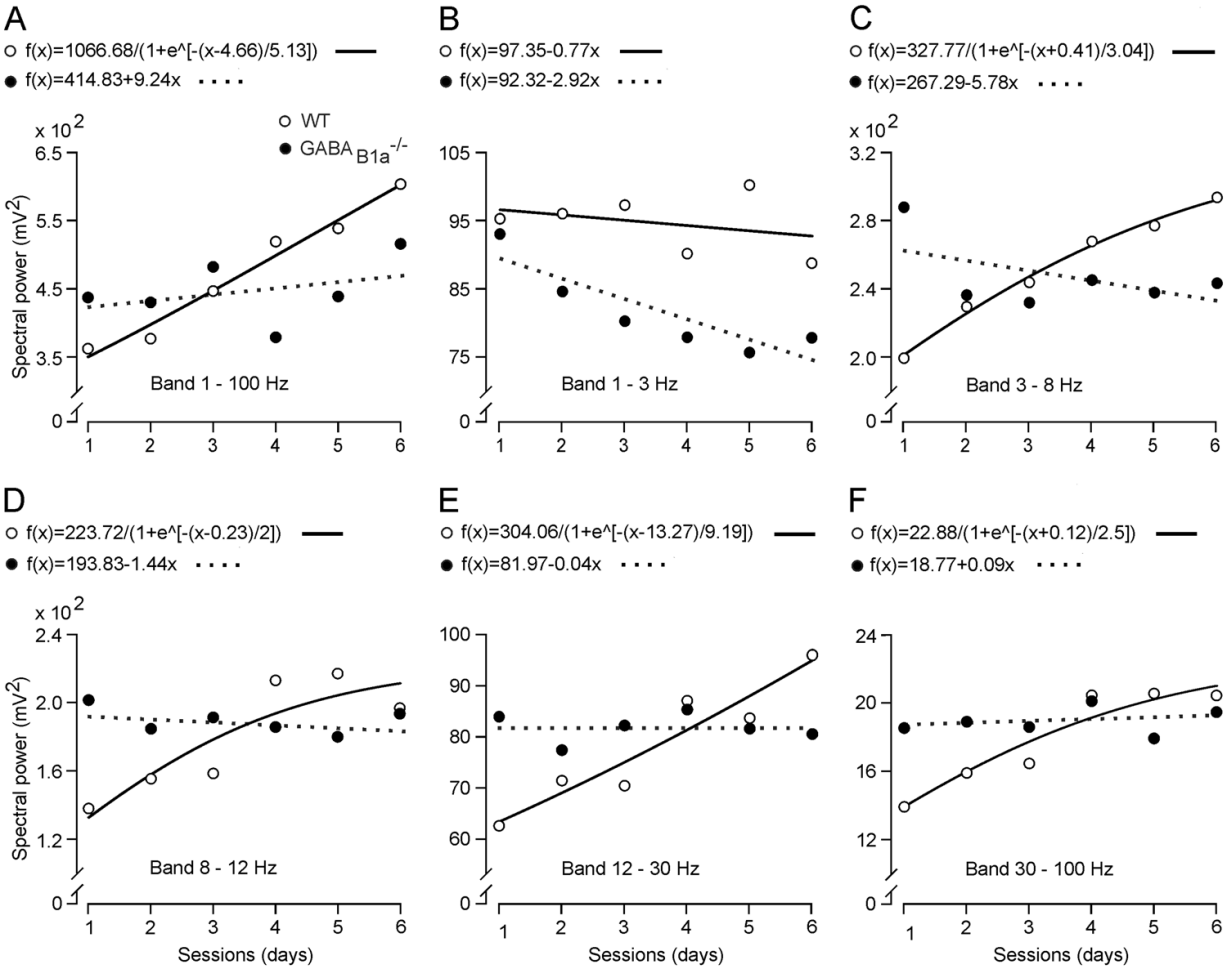
LFP changes in the CA1 area during learning sessions using 1:1 ratio schedule. (*A-F*) Mean peak values (circles) and best fits (lines) of the spectral power corresponding to LPFs recorded during each learning session in WT (open circles and continuous lines) and GABA_B1a_^-/-^ (closed circles and dotted lines) mice for all spectral bands in *A* (1–100 Hz), and for selected spectral bands in *B-F* (1–3 Hz in *B*, 3–8 Hz in *C*, 8–12 Hz in *D*, 12–30 Hz in *E* and 30–100 Hz in *F*). Note that peak spectral power values across the 3–8 Hz to the 30–100 Hz bands (*C-F*) tend to increase with each training session in WT mice, while spectral power values decreased or remained unchanged in GABA_B1a_^-/-^ mice. Changes in peak spectral power across the learning sessions reflect physiological changes during the learning sessions. The linear or non-linear equations that best fitted (*P* ≤ 0.05) spectral power values as a function of session number are shown above the corresponding graphs.

We analyzed the electrical activity of the CA1 pyramidal layer during the first 6 training sessions of the operant conditioning task (1:1 ratio). We initially collected electroencephalographic epochs that each lasted 1 s in the absence of electrical stimulation of Schaffer collaterals. The averaged (n = 20 times) spectral power of the local field potential (LFP) activity recorded during the 6 sessions is shown in Fig 2A. The spectral power of LFPs collected from WT mice increased in magnitude across the training for most spectral bands (3–8 Hz, 8–12 Hz, 12–30 Hz, and 30–100 Hz; Fig 2C–2F), as previously observed with a similar operant conditioning task [17]. In contrast, GABA_B1a_^-/-^ mice did not exhibit any increase in spectral power (8–12 Hz, 12–30 Hz, and 30–100 Hz; Fig 2D–2F) or showed a significant decrease (*P* < 0.05) for some spectral bands (1–3 Hz and 3–8 Hz; Fig 2B and 2C). Thus, deficits of GABA_B1a_^-/-^ mice in the acquisition of the operant conditioning task are paralleled by a deficit in the synchronization of neuronal activity in the hippocampal CA1 area during operant conditioning.

### Input/Output Curves and Short-Term Plasticity at CA3-CA1 Synapses of GABA_B1a_^-/-^ Mice Appear Normal

**Fig 3:**
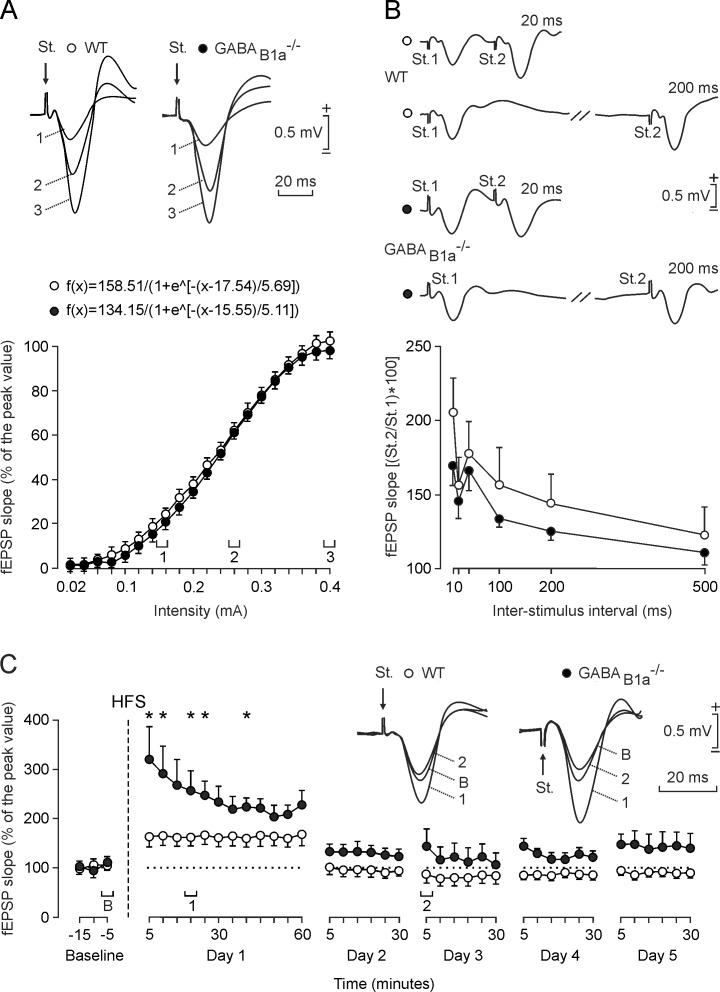
GABA_B1a_^-/-^ mice exhibit increased LTP of evoked fEPSPs in the CA1 area while input/output curves and paired-pulse facilitation are normal. (*A*) Input/output curves for the CA3-CA1 synapse. A single (100 μs, biphasic) pulse was presented to Schaffer collaterals at increasing intensities (from 0.02 mA to 0.4 mA, in steps of 0.02 mA) while recording evoked fEPSPs in the CA1 area for WT (open circles) and GABA_B1a_^-/-^ (closed circles) mice. Representative fEPSPs recorded from the stratum radiatum evoked with the intensities indicated in the graph (1, 2, 3) are shown at the top for each genotype. Equations corresponding to the best (*r* ≥ 0.996; *P* < 0.0001) sigmoid fits of the data [mean ± SEM; n ≥ 8 mice and ≥ 40 measurements for each of the 20 different stimulus intensities applied] are indicated. (*B*) Paired-pulse facilitation at the CA3-CA1 synapse. The graph shows the slopes of the second fEPSPs expressed as a percentage of the first (mean ± SEM) for six inter-stimulus intervals (10, 20, 40, 100, 200, and 500 ms). WT and GABA_B1a_^-/-^ mice exhibited paired-pulse facilitation at intervals of 10–200 ms (*P* < 0.01) that was not significantly different between the genotypes (*P* = 0.342). Representative recordings at 20 ms (top) and 200 ms (bottom) of inter-stimulus interval are shown on top (open circles for WT and closed circles for GABA_B1a_^-/-^). (*C*) Time course of LTP in the CA1 area (fEPSP mean ± SEM) following HFS. The HFS was presented after 15 min of baseline recording, at the time marked by the dashed line. The fEPSP is given as a percentage of the baseline (100%) slope. WT and GABA_B1a_^-/-^ mice showed a significant increase (ANOVA, two-tailed) in fEPSP slope following HFS when compared to baseline at day 1 (*P* < 0.001). fEPSP slope values were significantly (*, *P* ≤ 0.03) larger for GABA_B1a_^-/-^ than WT mice during the 5 days of recording. fEPSPs collected from WT (open circles) and GABA_B1a_^-/-^ (closed circles) mice before (baseline, B) and after (1, 2) HFS of Schaffer collaterals are shown on top.

To study whether the above deficits of GABA_B1a_^-/-^ mice in operant learning and network synchronization are caused by alterations in the basic electrophysiological properties of CA3-CA1 synapses we quantified input/output curves. These experiments were carried out in the same two groups of mice. Single pulses of increasing intensity delivered to the ipsilateral Schaffer collaterals evoked similar slope increases in CA1 fEPSPs in WT and GABA_B1a_^-/-^ mice (Fig 3A). In both groups of mice the input/output relationships were best fitted by sigmoid curves (*r* ≥ 0.996; *P* < 0.0001), supporting that the CA3-CA1 synapse in alert GABA_B1a_^-/-^ mice functions normally.

We additionally tested whether a typical short-term plasticity phenomenon [22] at the CA3-CA1 synapse, synaptic facilitation, is altered in GABA_B1a_^-/-^ mice. Synaptic facilitation evoked by the presentation of a pair of pulses has been related to increased neurotransmitter release during the 2nd pulse [26]. WT and GABA_B1a_^-/-^ mice both showed a typical and significant [F_(1,17)_ = 254.284; *P* < 0.01] increase in the response to the 2nd pulse at short (10–200 ms) time intervals (Fig 3B). The increase in the response to the 2nd pulse had a tendency to be smaller with GABA_B1a_^-/-^ than with WT mice but this tendency did not reach statistical significance at any of the selected intervals [F_(1,17)_ = 0.956; *P* = 0.342]. It therefore appears that this form of short-term plasticity is normal in GABA_B1a_^-/-^ mice.

### Long-Term Potentiation (LTP) at CA3-CA1 Synapses Is Increased in GABA_B1a_^-/-^ Mice

We next studied LTP induced by high-frequency stimulation (HFS) of the CA3-CA1 synapse in alert mice (Fig 3C). GABA_B1a_^-/-^ and WT mice exhibited a robust LTP following HFS [F_(1,12)_ = 169.519; *P* < 0.001]. Selectively the GABA_B1a_^-/-^ mice also exhibited increased LTP during the next four days of recording. The LTP evoked in GABA_B1a_^-/-^ mice was significantly larger and longer-lasting than that of WT animals [F_(1,13)_ = 6.409; *P* ≤ 0.03] during the first recording day. A possible explanation for this finding is that disinhibited glutamate release in the GABA_B1a_^-/-^ mice facilitates the development of LTP [12].

### GABA_B1a_^-/-^ and WT Littermate Mice Differ in Oscillatory Properties of Hippocampal LFPs

**Fig 4:**
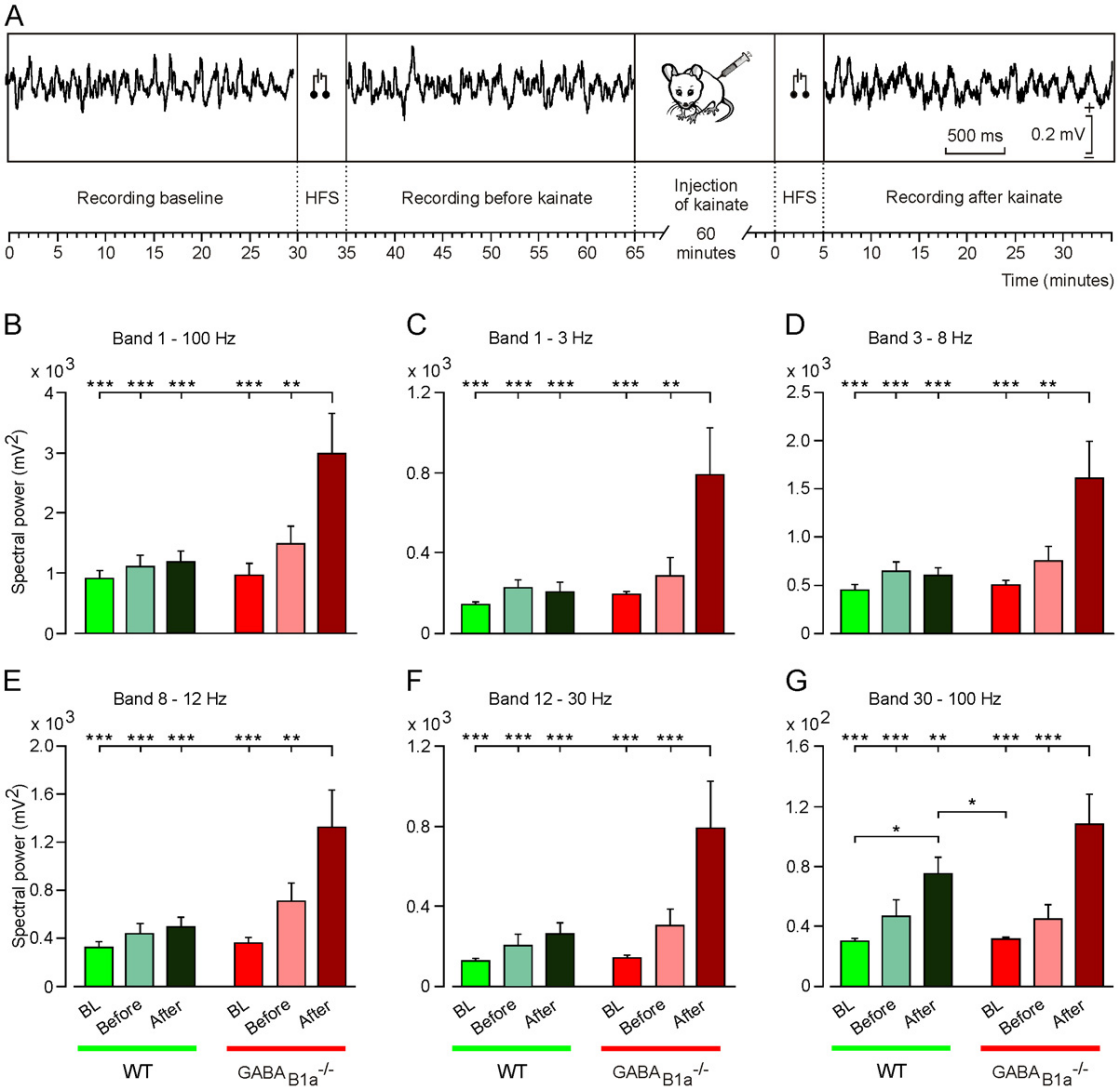
GABA_B1a_^-/-^ mice exhibit a drastic increase in the spectral power of LFP activity in the presence of kainate. (*A*) Scheme of the experimental protocol used. LFP activity in the CA1 area was recorded for 30 min to establish a baseline. Then, a HFS protocol (consisting of five 200 Hz, 100 ms trains of pulses at a rate of 1/s) was presented and the evoked LFP activity was recorded for another 30 min. After that, mice were injected with kainate (8 mg/kg), and 60 min later LFP activity was recorded again. Calibration for the selected LFP traces is indicated on the right. (*B-G*) Histograms representing the maximum values of the spectral power of LFP activity recorded during baseline (BL) recordings, and before and after kainate injection into WT (green histograms) and GABA_B1a_^-/-^ (red histograms) mice. Values of all spectral bands (1–100 Hz) are depicted in *B*, selected spectral bands in *C-G* (1–3 Hz in *C*, 3–8 Hz in *D*, 8–12 Hz in *E*, 12–30 Hz in *F*, and 30–100 Hz in *G*). Note that for all spectral bands the maximum values of the spectral power were seen in GABA_B1a_^-/-^ mice in the presence of kainate and the second HFS.*, *P* < 0.05; **, *P* < 0.01; ***, *P* < 0.001.

Presynaptic GABA_B_ receptors contribute to spontaneous down-state transitions in the entorhinal cortex [7]. GABA_B1a_^-/-^ mice of several months of age occasionally develop seizures [15]. It is therefore surprising that GABA_B1a_^-/-^ mice fail to synchronize neuronal activity in the hippocampal CA1 area during operant conditioning. We decided to study the oscillatory properties of LFPs in behaving GABA_B1a_^-/-^ mice to address whether these mice suffer from a general deficit in synchronizing network oscillations. Under baseline conditions, GABA_B1a_^-/-^ and WT littermate mice presented LFPs in the CA1 area of similar spectral power. A brief HFS (five 200 Hz, 100 ms trains of pulses at a rate of 1/s) applied to the Schaffer collaterals evoked a slight but statistically non-significant increase in the spectral power compared to baseline in GABA_B1a_^-/-^ mice, an effect that was barely detectable in WT mice (Fig 4A and 4B).

The proconvulsive drug kainate has been used to determine the propensity of cortical circuits to oscillate [25]. When we applied the same brief HFS protocol in the presence of a low dose (8 mg/kg) of kainate we observed a significant increase in spectral power with GABA_B1a_^-/-^ but not with WT mice (Fig 4A and 4B). Analysis of frequency bands in the presence of kainate revealed a significantly increased spectral power in GABA_B1a_^-/-^ mice across the entire LFP spectrum (Fig 4C–4G). Of note, HFS in the presence of kainate also induced a significant increase in spectral power in WT mice for the gamma band (30–100 Hz; Fig 4G).

**Fig 5:**
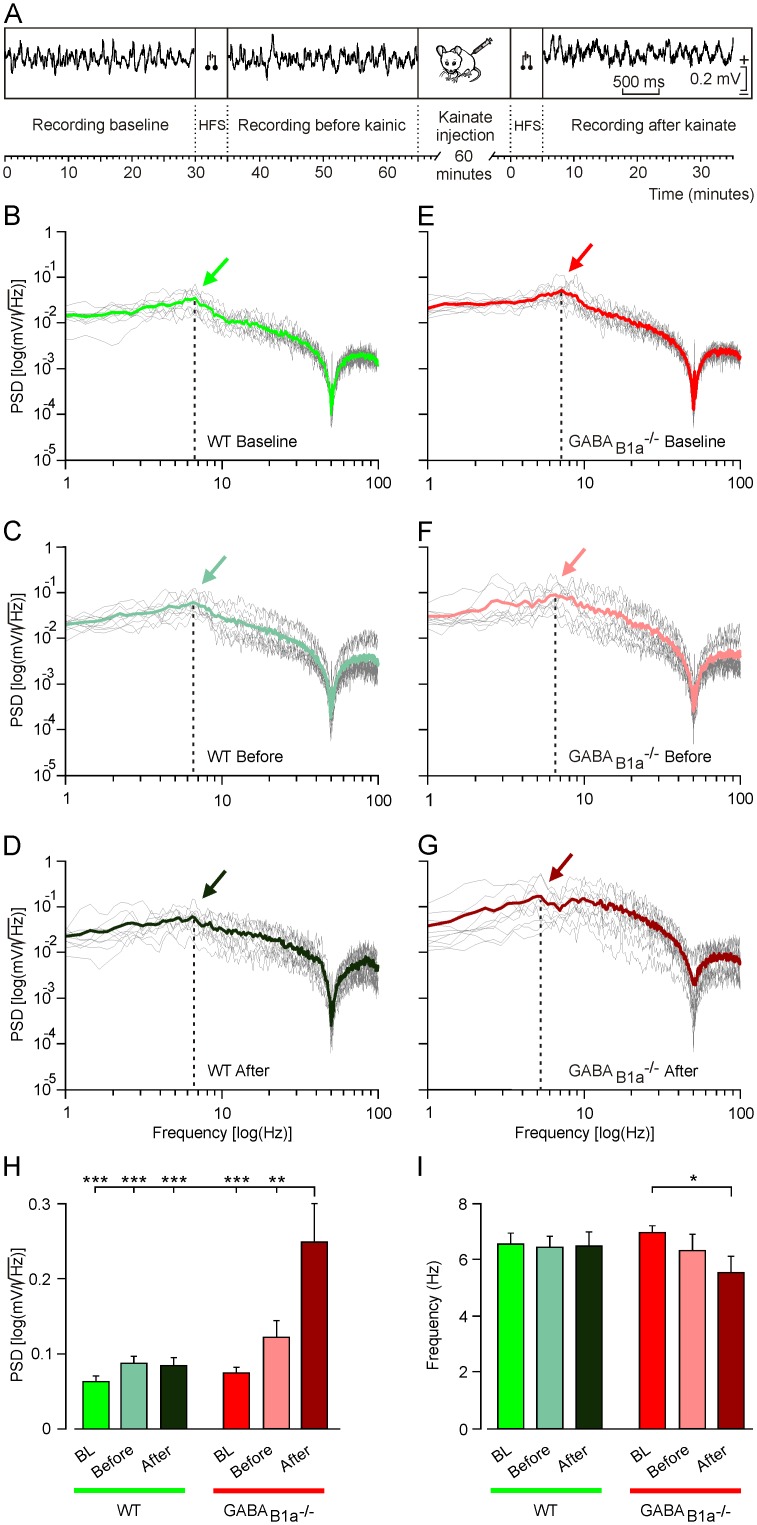
Differences in the PSD of LFPs recorded in the hippocampal CA1 area during different states of neuronal activation. (*A*) Scheme of the experimental protocol used. (*B-G*) PSD plots for the LFP activity recorded during baseline (WT, in *B*; GABA_B1a_^-/-^, in *E*), and before (WT group, in *C*; GABA_B1a_^-/-^ group, in *F*) and after (WT group, in *D*; GABA_B1a_^-/-^ group, in *G*) kainate injection (8 mg/kg). Colored arrows in *B-G* indicate the dominant frequency of the spectra [PSD values, in log (mV/√Hz) are indicated on the y-axis; corresponding frequencies, in log (Hz), are indicated on the x-axis]. The mean values of the represented traces are indicated by the colored traces. (*H*) Histograms representing PSDs corresponding to each group and the three different recording situations [baseline (BL, green and red histograms), and before (light green and light red histograms) and after (dark green and dark red histograms) kainate administration]. (*I*) Histogram representing the dominant frequency of the LFP activity recorded during baseline (BL, green and red histograms), and before (light green and light red histograms) and after (dark green and dark red histograms) kainate injection for both genotypes. Note in *H* and *I* that the maximum PSD value and the minimum value of the fundamental frequency was recorded in the GABA_B1a_^-/-^ group following HFS after injection of kainate. For both *H* and *I*, collected data was quantified for each experimental animal (n = 5 per group) and then averaged. *, *P* < 0.05; **, *P* < 0.01; ***, *P* < 0.001.

Analysis of the PSD allowed us to determine the spectral power and periodicity of the LFP data by observing the power peak at the frequency corresponding to that periodicity (Fig 5A). PSD plots (Fig 5B–5G) for the LFP activity revealed that HFS, in particular in the presence of kainate, induced a significant increase of the PSD value (Fig 5H) and a shift of the dominant frequency towards lower frequency in the GABA_B1a_^-/-^ mice (6.97 Hz before HFS, 6.36 Hz after HFS, and 5.54 Hz after kainate + HFS; Fig 5E–5G and 5I), a change that was absent in WT mice (6.56 Hz, 6.46 Hz, and 6.51 Hz respectively; Fig 5B–5D and 5I).

In summary, the GABA_B1a_^-/-^ mice differ from WT mice in their propensity to synchronize electrocortical activities upon HFS in the presence of kainate. It is therefore counterintuitive that GABA_B1a_^-/-^ mice are unable to appropriately synchronize network activity during associative learning tasks.

## Discussion

Ultrastructural methods revealed that GABA_B_ receptors in the hippocampus are most abundant at glutamatergic terminals [2], [27], [28]. We previously used pharmacological and physiological activation of GABA_B_ receptors in acute slices to show that selectively GABA_B(1a,2)_ receptors inhibit glutamate release at brain synapses, including the CA3-CA1 and mossy fiber-CA3 synapses [2], [12], [29]. Lack of GABA_B(1a,2)_ receptors affects the expression of LTP and produces cognitive deficits [12], [30]. However, it remained unclear whether behavioral stimuli can activate GABA_B(1a,2)_ receptors. GABA released from GABAergic terminals has to diffuse to neighboring synapses to activate GABA_B(1a,2)_ receptors at glutamatergic terminals. Interneurons therefore have to fire in synchrony to release sufficient GABA to spill over to glutamatergic terminals. In keeping with a requirement for synchronous interneuron activity to engage GABA_B(1a,2)_ receptors these receptors exert a powerful inhibitory influence over network oscillations *in vitro* [7]. Cortical neurons display synchronous fluctuations between periods of persistent activity ('UP states') and periods of relative quiescence ('DOWN states'). Presynaptic GABA_B(1a,2)_ receptors contribute to spontaneous DOWN state transitions in slice preparations of the entorhinal cortex [7]. Consistent with a role for GABA_(B1a_,_2)_ receptors in the control of network oscillations, GABA_B1a_^-/-^ mice also have a propensity to develop seizures at an advanced age [15].

Thus far, only one study has addressed whether sensory stimuli can activate GABA_B(1a,2)_ and/or GABA_B(1b,2)_ receptors at identified synapses. Using anesthetized GABA_B1b_^-/-^ mice, Palmer and colleagues found that postsynaptic GABA_B_ receptors exert an inhibitory influence on cortical layer 5 pyramidal cells in response to hind paw stimulation [27]. This supports that salient sensory events recruit postsynaptic GABA_B(1b,2)_ receptors.

Here we used behaving GABA_B1a_^-/-^ mice with implanted electrodes to address whether presynaptic GABA_B_ receptors in the hippocampus are recruited during an operant conditioning task. As already described, both the hippocampus [17] and prefrontal cortical areas [18] participate in the acquisition of this type of associative task. We found that GABA_B1a_^-/-^ mice exhibit a deficit in learning-dependent increases in synaptic strength at hippocampal CA3-CA1 synapses and a lack of network synchronization during the acquisition phase. Interestingly, the largest increase in fEPSP slopes observed with WT mice took place one session before reaching the criterion. The fEPSP slopes then remained above baseline values for the remaining sessions (3 to 6). In a previous report we observed the largest increase in fEPSP slopes one session after reaching the criterion [17]. A possible explanation for this discrepancy is the genetic background of the mice used in the two studies. The BALB/c mice used in the present study appear to learn faster than the C57BL/6 mice used in the earlier study [17], consistent with a report that compared the two mouse strains in learning tasks [31].

These findings were paralleled with impairment in the acquisition of the task. Of note, basic properties of CA3-CA1 synapses, such as paired-pulse facilitation and input-output relationships, were normal in behaving GABA_B1a_^-/-^ mice, supporting that these synapses do not undergo major adaptive changes. Our results therefore support that activation of presynaptic GABA_B_ receptors in the hippocampus is necessary during the acquisition of a learning task and for learning-dependent synaptic changes and network dynamics. A previous *in vitro* study in acute slices of 3-week old mice, revealed a deficit in LTP at CA3-CA1 synapses, which was explained by a saturation of LTP mechanisms due to uncontrolled glutamate release [12]. In contrast LTP at CA3-CA1 synapses in response to HFS was increased in behaving GABA_B1a_^-/-^ mice. The present findings may be explained by differences in the LTP protocols (40 stimuli at 0.05 Hz paired with postsynaptic depolarization versus HFS) or homeostatic mechanisms that, in older mice, drift the synapse back into the dynamic range. It is also possible that the HFS protocol used here evokes NMDA receptor-independent LTP in the CA1 region [32], which is not the case with the LTP protocol used during *in vitro* experiments [12].

Increased LTP in response to HFS in GABA_B1a_^-/-^ mice also contrasts with a lack of learning-dependent increases in synaptic strength. A similar dissociation between HFS-induced CA3-CA1 synaptic plasticity and a lack of changes in synaptic strength during associative learning has been described in transgenic mice overexpressing the neurotrophin receptor TrkC [33] or the transcription factor CREB [34]. This dissociation may be explained by a differential activation of interneurons during HFS and instrumental conditioning. GABA_B1a_^-/-^ mice do not show the typical network synchronization during acquisition of the instrumental conditioning task. This cannot be explained by a general inability of behaving GABA_B1a_^-/-^ mice to synchronize network oscillations in the hippocampus, since these mice show an increased propensity to synchronize their electrocortical activity in the presence of kainate after HFS. It appears that presynaptic GABA_B(1a,2)_ receptors are necessary to induce learning-dependent changes in network dynamics while kainate more efficiently induces network synchronizations through postsynaptic glutamate receptors when presynaptic GABA_B(1a,2)_ receptors are absent. This suggests that the (homeostatic) balance of excitation/inhibition at pre- and postsynaptic sites is crucial for inducing learning-dependent network synchronizations and/or to prevent the development of an excessive synchronization leading to the appearance of unwanted seizures [35]. In summary, our experiments show that deficits in presynaptic GABA_B(1a,2)_ receptor signaling lead to perturbed network activity, which not only impairs normal learning but ultimately also manifests as epilepsy [15].
